# Supplementary material for: Are carbon emissions trading and green financial instruments synergistic? -Comprehensive quantitative research based on content analysis
Source: PLoS One. 2024 Mar 7;19(3):e0298601. doi: 10.1371/journal.pone.0298601 (PMC10919601; doi:10.1371/journal.pone.0298601)
Supplement: S1 Data — (ZIP) [file pone.0298601.s001.zip › Data/地级市Z_F工作报告.zip!/░▓╗╒/║╧╖╩╩╨/└┤╫╘╣1⁄2╓┌║┼í╛┬φ┐╦╩2╛▌═°í┐_2019.docx]

# **2019年政府工作报告**

发布时间：2019-01-08 15:09

【字体：[大](http://www.hefei.gov.cn/public/1741/javascript:void(0);) [中](http://www.hefei.gov.cn/public/1741/javascript:void(0);) [小](http://www.hefei.gov.cn/public/1741/javascript:void(0);)】

各位代表：

　　现在，我代表市人民政府向大会报告工作，请予审议，并请政协委员和其他列席人员提出意见。

　　一、2018年工作回顾

　　2018年，在省委省政府和市委的坚强领导下，我们全面贯彻落实习近平新时代中国特色社会主义思想和党的十九大精神，主动对标高质量发展要求，坚持稳中求进工作总基调，大力实施五大发展行动计划，攻坚克难，锐意进取，较好完成了市十六届人大一次会议确定的各项目标任务，实现了本届政府良好开局。

　　预计，全市生产总值增长8.5%；财政收入1378.3亿元、增长10.2%，其中地方财政收入712.5亿元、增长8.6%；规模以上工业增加值增长11.2%；固定资产投资增长7%；社会消费品零售总额增长10%；完成进出口总额300亿美元以上、增长20%；城镇登记失业率2.9%；节能减排完成省控目标。

　　——创新驱动跑出加速度。创新型城市建设取得重大进展，“四个一”创新主平台加快培育，合肥综合性国家科学中心扎实推进，滨湖科学城拉开框架，全社会研发投入占GDP比重达到3.24%，入选全球科研城市50强，“大力培育创新平台、助力产业发展”典型经验获得国务院通报表彰，9项成果荣获2018年度国家科学技术奖。

　　——区域发展创造新业绩。肥西、肥东、长丰在全国百强县位次前移，巢湖入选全国首批创新型县（市），庐江再次跻身全国投资潜力百强县。包河、蜀山、庐阳、瑶海巩固全国综合实力百强区地位。高新区跻身世界一流高科技园区建设序列，经开区跃居国家级经开区产业基础前十，新站高新区加快打造世界级新型显示产业基地，安巢经开区扎实推进中国创新力百强园区及国家级旅游度假区建设。

　　——生态建设取得新进展。蓝天、碧水、净土保卫战全面展开，PM10、PM2.5平均浓度下降8.8%、14.3%，连续5年实现“双下降”，空气质量明显改善。地表水15个国考断面中13个达标、1个达到国家考核要求，水环境质量持续好转。土壤污染状况调查全面展开，城区4个地块土壤修复稳步推进。

　　——改革开放实现新突破。全面深化改革，“全创改”、“放管服”、国资国企、农业农村、财税金融等重要领域改革稳步落实，市场主体活力持续释放。深度融入“一带一路”建设和长江经济带、长三角一体化发展，大平台大通道大通关提速建设，首届世界制造业大会和2018国际徽商大会成功举办，安徽省庆祝改革开放40周年科技创新成果展精彩圆满。

　　——人民群众增添幸福感。实打实办好31项民生工程和20件民生实事，全市民生支出859.8亿元、占财政支出85.6%。城镇新增就业突破25万人，居民收入增长快于经济增长，城镇、农村居民人均可支配收入分别达到41300元、20200元，增长9%左右。脱贫攻坚取得决定性进展。

　　一年来，我们主要做了以下工作：

　　（一）瞄准前沿，创新动能持续增强。大科学装置和重大创新平台建设取得积极进展，国际聚变能联合中心正式成立，聚变堆主机关键系统园区顺利开工，量子创新院、中科大高新园区全面建设，安徽创新馆、离子医学中心、类脑实验室、中国声谷等标志性项目有序推进，8个省实验室和7个省技术创新中心组建运行。中德教育合作示范基地、清华公共安全研究院、合肥北航科学城等协同创新平台加快建设。合肥国际人才城建成使用，国家海外人才离岸创新创业基地启动建设，合肥成为“外籍人才眼中最具吸引力的中国城市”。新增国家高新技术企业400多户、总数突破2000户，全市发明专利授权量达到5700件、同比增长15%。全超导托卡马克装置首次实现1亿摄氏度等离子体运行，中科大18个量子比特纠缠刷新世界纪录，首款国产量子计算机控制系统在肥诞生，稳态强磁场、同步辐射等一批国之重器在科学星海中闪耀光芒。一年来，我们坚持下好创新“先手棋”，集聚创新要素，激发创新活力，创新之都正昂首迈向高光时刻。

　　（二）聚焦高端，产业升级持续加快。出台推动经济高质量发展30条政策，安排100亿元资金扶持实体经济发展，民营经济发展考核连续11年荣获全省第一。深入实施“三重一创”，统筹推进战新产业集聚发展基地建设，智能语音基地入列首批国家先进制造业集群培育试点，战新产业增加值增长18%左右、对全市工业增长贡献率达70%以上。京东方10.5代线、晶合12吋晶圆、通威2.3GW高效晶硅电池、江汽蔚来等项目投产量产，江淮大众首款新能源汽车“思皓”正式下线，维信诺第六代柔性显示生产线开工建设。家电、装备制造等传统优势产业加速智能化改造，建成57家智能工厂、495个数字化车间。服务业集聚区发展五年规划出台实施，高科技服务、文化创意、旅游休闲等新业态迅猛发展，合肥数字经济位居省会城市前十，新能源汽车博览会、家博会、农交会、苗交会等展会影响力、美誉度持续提升，区域性金融中心功能不断增强。合肥电子信息军民融合产业基地获批建设，省级军民融合服务平台正式落户。

新兴产业和传统产业共同发力，先进制造业和现代服务业加快融合，“合肥制造”正加速向全球价值链中高端迈进。一年来，我们坚持服务实体经济，聚力产业创新，推动“三个变革”，培植起高质量发展的厚实根基。

　　（三）攻坚突破，改革开放持续发力。以优化环境、提升效能为着力点，“多证合一”“证照分离”改革全面展开，“一网一门一次”改革取得重要突破，在全省率先实现企业开办3日、不动产登记5日办结，新登记市场主体20万户。实施国有资产分类监管，国有资本引领产业升级、保障公共服务能力显著提升。农村承包地确权登记颁证全面完成，306个村居完成“三变”改革。医疗卫生和医保管理体制改革有序推进，市、县两级医改（医保）管理机构全部挂牌成立。开放平台功能持续提升，合肥中欧班列开行182列、增长1.6倍；水运港集装箱吞吐量突破30万标箱；空港保税物流中心（B型）封关运行，进境食用水生动物口岸开检；中国（合肥）跨境电商综试区保税、直购进口业务开通；出口加工区进出口额位居全国第3。招大引强成果丰硕，完成招商引资3000亿元、增长9.1%。对外合作交流取得新进展，荣获“国际友好城市交流合作奖”，获批全国首家“海峡两岸集成电路产业合作试验区”。深化与长江经济带城市合作，加入长三角G60科创走廊。出台合肥都市圈一体化发展行动纲要，加强与皖北结对合作，合阜产业园在全省援建考评中名列第一。一年来，我们坚持推进改革开放，以改革增动力，以开放添活力，展现海纳百川的开放格局。

　　（四）提质提品，城市功能持续完善。提升全国性综合交通枢纽地位，商合杭、合安高铁加快建设，新合肥西站完成设计方案，南站南广场工程主体结构完工；滁淮高速、集贤路互通等竣工通车，德上高速合枞段、岳武高速东延无岳段开工建设。引江济淮工程全面建设。启动编制新一轮城市总体规划，完成东部新中心、空港国际小镇等重点片区规划。持续推进大建设，新建续建工程1248项，完成投资529亿元。城市轨道交通1、2号线日均客流达42万人次，扫码乘车成为现实；3、4、5号线和1号线三期工程进展顺利。铜陵路、阜阳路高架北延、长江西路快速化改造建成通行，南淝河路、龙岗路等开工建设，首批12个城市拥堵点治理工程全部完工。建成公共停车泊位5348个、充电桩9582个，公交都市创建深入推进。长江中路、科学岛路等一批精品道路加快实施，金寨路高架等景观亮化工程顺利完工。开展城市“大综管”试点，数字城管水平显著提升。一年来，我们坚持提升城市品质，在有温度中建设，在求精度中管理，打造宜居宜业的美好家园。

　　（五）统筹协调，城乡融合持续深化。扎实开展脱贫攻坚“重精准、补短板、促攻坚”专项行动，不断提高精准脱贫质量，“十大工程”省定任务全面完成，全年减少贫困人口2477人。实施乡村振兴工程，一批重点项目稳步推进。现代都市农业发展步伐加快，新增新型农业经营主体1400家，“三品一标”发展到957个，长丰草莓成功入选中国特色农产品优势区。粮食产量突破300万吨，虾稻综合种养面积突破20万亩，肉蛋奶及水产品总量超过93万吨。美丽乡村建设扎实推进，完成70个中心村建设，改造农村危房3703户。农村环境“三大革命”纵深推进，建成18个乡镇政府驻地污水处理设施，超额完成10万户农村改厕任务，生活垃圾无害化处理率达到90%以上。建成农村道路畅通工程2379公里，肥西成功创建“四好农村路”全国示范县。大力实施土地整治，连续20年实现占补平衡。全面完成农村安全饮水建设任务，解决26.8万农村居民饮水安全问题。一年来，我们坚持城乡融合发展，推动以城带乡，促进城乡一体，全面开启乡村振兴的新征程。

　　（六）标本兼治，生态环境持续改善。制定坚决打好污染防治攻坚战实施意见、水清岸绿产业优美丽长江（合肥）经济带实施方案、巢湖综合治理绿色发展总体规划，狠抓中央和省环保督察问题整改。环巢湖生态保护与修复工程顺利推进，建成胡大郢、于湾污水处理厂，完成城区污水处理厂提标改造。严格落实“河长制”“湖长制”，创新实施“排长制”，新建雨污管网559公里，双桥河、派河2条重污染河流消除劣Ⅴ类。突出抓好烟花爆竹禁燃、餐饮油烟专项整治，空气质量明显改善，优良天数比率达到74%、同比提高12.3个百分点。在全省率先开展地表水、空气质量生态补偿试点。城市十大公园和环湖十大湿地启动建设，四级“林长制”实现全覆盖，全年植树造林6万多亩、城镇绿化1377万平方米，新增绿色长廊240公里，新建花境528处。一年来，我们坚持践行绿色发展理念，实施铁腕治污，坚守生态底线，绘就蓝天白云、水清岸绿的美丽画卷。

　　（七）普惠共享，民生福祉持续提升。用心办好人民满意的教育，深入推进义务教育“三大提升”工程，实施特殊教育二期提升计划，建成中小学27所、幼儿园56所，市区中小学午餐服务工程覆盖率超过98%。合肥四中、合职院汇心湖校区、市公共实训中心等投入使用，黄麓师范学校改扩建一期建设完工，北外德国中心（合肥）正式挂牌。合肥学院建设应用型大学、合肥幼专幼师培养模式改革创新、合肥六中数字实验研究推广等8项成果获2018年国家级教学成果奖。统筹县（市）城乡低保标准，整合城乡居民基本医疗保险、大病保险、医疗救助政策，推进居家养老服务三级中心建设。国家健康医疗大数据中部中心及产业园启动建设，滨湖智慧医院一期建成。促进房地产市场健康发展，稳步推进住房租赁试点；新开工棚户区改造安置房超过2万套，综合整治老旧小区173个。新建57个城市阅读空间，成功举办第十届中国曲艺牡丹奖大赛（合肥赛区）、第十二届合肥国际文博会等一批重大文化活动，现代舞《命运》、小品《等爱回家》等荣获国家级大奖。建成体育健身场所173个，成功举办合肥国际马拉松等赛事，省运会实现金牌总数等六项第一。全力创建国家食品安全示范城市，城区一半以上存量菜市场完成改造升级。深入推动安全生产“铸安”行动常态化实效化，安全生产形势稳定向好。“雪亮工程”示范城市建设加快推进，扫黑除恶专项行动成效明显。市信访事项人民调解委员会正式挂牌，信访工作机制不断健全。深入开展“七五”普法，公共法律服务体系日趋完善。在全国首创“五社联动”社区治理服务新机制。成立退役军人事务局，国防教育、国防动员、人民防空、双拥优抚安置工作成效显著。民族宗教、防震减灾、地方志、统计、科普、气象、档案、保密等工作水平实现新提升，工会、共青团、妇女儿童、红十字会、老年人、残疾人和关心下一代等工作取得新进步。一年来，我们坚持改善民生福祉，解百姓之忧，办百姓之需，努力交上一份人民满意的民生答卷。

　　过去一年，我们准确把握新时代党的建设总体要求，深入推进“两学一做”学习教育常态化制度化，扎实开展“讲严立”专题警示教育和“不忘初心牢记使命、做好表率走在前列”大调研，不断提升政府自身建设水平。自觉接受人大监督，严格执行人大及其常委会的决议决定，主动接受政协民主监督、社会监督和舆论监督，全年办理人大代表议案和建议280件、政协提案510件。强化依法行政，法治政府建设始终走在全国全省前列。大力推进“互联网+政务服务”平台建设，获评全国十大“办事不跑腿”城市、2018营商环境省会十佳城市。严格落实党风廉政建设责任制，驰而不息正风肃纪，持续强化审计监督，廉洁政府建设取得新成效。

　　各位代表！

　　过去一年，我们在奋力攻坚中排除万难，我们在上下同心中增添信心。合肥发展稳中向好、稳中提质、稳中育新，成绩来之不易。这是省委省政府和市委坚强领导的结果，是市人大依法监督、市政协民主监督、社会各界大力支持的结果，是全市人民团结奋斗、顽强拼搏的结果。在此，我代表市人民政府，向辛勤奋战在各个领域、各个岗位为合肥发展作出贡献的全市人民，向驻肥人民解放军、武警官兵、公安干警、消防救援队伍指战员和中央驻肥单位，向各民主党派、工商联、无党派人士、各人民团体和社会各界人士，向所有关心支持合肥改革开放与现代化建设的海内外朋友，表示衷心的感谢和崇高的敬意！

　　在肯定成绩的同时，我们也清醒看到合肥发展中存在的不平衡、不充分问题，主要是：支撑高质量发展的新动能仍然不足，民营企业和中小微企业融资难融资贵问题依然突出；经济结构有待进一步优化，现代服务业总体发展不足；巢湖综合治理、大气污染防治等任务艰巨，群众身边环境问题依然较多；大城市管理水平有待提升，文明城市创建仍需巩固；优质教育、医疗、养老、文化等公共服务供给与群众期盼还有差距；效能建设仍需加强，少数部门和工作人员服务能力不强、创新办法不多等问题不同程度存在。对此，我们将采取切实有效措施，认真加以解决。

　　二、2019年重点任务

　　今年是新中国成立70周年，是完成“十三五”发展规划目标和全面建成小康社会的关键之年，做好今年工作意义重大。我们必须瞄准新坐标、彰显新担当。树立全球视野、战略思维，对标先发城市，始终以领跑者的姿态，中流击水，奋楫争先，在新一轮世界科技革命和产业变革中抢占先机、加快转型，在全国区域发展新格局中提升能级、凸显地位。我们必须把握新时代、践行新理念。自觉践行新发展理念，加快形成创新成为第一动力、协调成为内生特点、绿色成为普遍形态、开放成为必由之路、共享成为根本目的的发展模式，始终以追赶者的姿态，积极应对更趋严峻复杂的外部环境，努力实现更高质量、更有效率、更加公平、更可持续的内涵式发展。我们必须顺应新期待、展示新作为。坚持以人民为中心的发展思想，把人民对美好生活的向往，落实到能获得、有感受的民生实践中，始终以奋斗者的姿态，多谋民生之利、多解民生之忧，倾心尽力解决好交通出行、住房保障、环境改善和育幼、上学、看病、养老、就业等问题，让群众拥有更多获得感、幸福感、安全感。

　　今年政府工作的总体要求是：以习近平新时代中国特色社会主义思想为指导，全面贯彻落实党的十九大精神，统筹推进“五位一体”总体布局，协调推进“四个全面”战略布局，按照省委省政府和市委决策部署，坚持稳中求进工作总基调，以供给侧结构性改革为主线，坚持新发展理念，高质量推进五大发展行动，坚决打好三大攻坚战，全力做好稳增长、促改革、调结构、惠民生、防风险工作，进一步稳就业、稳金融、稳外贸、稳外资、稳投资、稳预期，保持经济持续健康发展和社会大局稳定，加快建设长三角世界级城市群副中心，奋力打造具有国际影响力的创新之都。

　　今年发展的主要预期目标是：全市生产总值增长8%；规模以上工业增加值增长9%；固定资产投资增长7%；财政收入增长6%；社会消费品零售总额增长9%；居民人均可支配收入增长快于经济增长；居民消费价格涨幅控制在3%以内；城镇新增就业13万人，城镇登记失业率控制在3%以内；节能减排完成省控目标。

　　（一）高定位建设国家科学中心。以综合性国家科学中心建设为引领，深入实施创新驱动发展战略，加快新旧动能转换，推动科技创新成为高质量发展的强大引擎。

　　建设一流平台。围绕“四个新高地”定位，高标准规划建设合肥滨湖科学城，强力打造“四个先行区”。大力构建综合性国家科学中心“2+8+N+3”创新体系，加快聚变堆园区和国际聚变能联合中心建设，争创量子信息科学国家实验室和国家智能语音创新中心，开工建设合肥超算中心，推进合肥先进光源、大气环境立体探测等预研。强化与中科大等高校院所合作，促进市校深度融合，主攻应用技术转化，建设开放型协同创新平台，让创新之源持续奔涌。

　　融合高端资源。开展基础前沿领域和重点产业核心技术攻关，争取国家“科技创新2030重大项目”落户，实施重大研发、科技小巨人项目50个。强化企业创新主体培育，新增国家高新技术企业不少于200户。开展产学研融合创新，新建重点实验室、企业技术创新中心等各类研发机构100家以上。鼓励创新主体加大投入，力争全社会研发投入增长10%以上，发明专利拥有量增长15%以上。建成安徽创新馆，举办科技创新成果博览交易会，让创新主体茁壮成长。

　　优化创新环境。深入推进“全创改”，构建良好创新生态。抢抓科创板试点注册制机遇，培育一批高新技术企业上市科创板。建设国家特色双创基地，打通从众创空间、工业园区到产业集群的全生命周期创新链，提升专业孵化水平。落实知识产权保护制度，创建中国（合肥）知识产权保护中心，建立优势产业和重点企业专利快速申请授权绿色通道。强化高端人才引进培育集聚，深入实施“6311”重点工程，用好首席科学家制度、科研人员股权激励等政策，打造人才“强磁场”，让创新活力竞相迸发。

　　（二）高质量推动产业创新。深化供给侧结构性改革，坚决贯彻落实“巩固、增强、提升、畅通”八字方针，推动先进制造业与现代服务业“双轮驱动”，全面提升产业核心竞争力。

　　推动先进制造业高质量发展。大力推进工业强基工程、“振芯铸魂”计划、技术改造行动，加快解决关键核心技术“卡脖子”问题。发挥国家、省、市三级战新集聚发展基地和省级工程（专项）引领作用，着力提升智能家电、集成电路、新型显示、智能语音、新能源汽车等产业集群国际竞争力，争创制造业高质量发展国家级示范区和国家产业创新中心。加快维信诺第六代柔性显示生产线、联宝新增年产千万台智能产品等重大产业项目建设，推动江淮大众新能源汽车、长安汽车二期等项目投产量产。强力推进数字经济发展，培育壮大以中国声谷为核心的人工智能产业，打造“数字化车间”“智能园区”，力争完成300个重点技术改造项目，培育15户智能工厂和180个数字化车间，实现5000户企业与云资源深度对接，让制造业成为合肥发展的坚强基石。

　　推动现代服务业加快发展。以服务业集聚区和示范园区建设为突破口，推动生产性服务业专业化发展，促进生活性服务业精细化发展。大力推进合肥国家综合物流枢纽、中国快递示范城市建设，加快庐阳金融城、滨湖金融小镇、派河国际物流园、高新区国家知识产权集聚区等一批重点项目建设，积极争创国家检验检测高技术服务业集聚区。做大做强文化旅游产业，创建巢湖·姥山岛国家5A级景区，推进巢湖市、庐江县创建国家全域旅游示范区。办好世界制造业大会、家博会等知名会展，全方位提升合肥的美誉度和影响力。推动互联网与传统零售业、家政服务等深度融合，加快孕育平台型消费、共享经济等新模式，激发居民消费潜力，促进消费提档升级，让服务业成为合肥发展的重要力量。

　　推动民营经济健康发展。制定实施民营经济发展五年行动计划，修订《合肥市促进民营经济发展条例》实施细则，全面落实国家和省各项减税降费政策，进一步降低企业社保缴费比例。设立民营经济发展专项资金，扩大新型政银担等普惠金融规模，培育更多“隐形冠军”“专精特新”企业。打造“四最”营商环境，常态化推进“四送一服”双千工程，为民营经济发展清障护航。保护企业和企业家合法权益，在执法过程中避免简单化，在政策执行中不搞“一刀切”，始终与民营企业站在一起，为自己人办事，让民营企业成为合肥发展的主力军。

　　（三）高品质加强城市建设。坚持人民城市为人民，优化城市空间布局，提升城市功能品质，推动城市发展由外延扩张式向内涵提升式转变，加快打造国际化都市区。

　　城市规划重引领。坚持世界眼光、国际标准、中国特色、高点定位的原则，统筹城乡生产、生活、生态三大空间，高质量编制完成新一轮城市总体规划，打造具有蓝绿交织、清新明亮、水城共融、多组团集约发展特色的美丽长三角世界级城市群副中心。加强重点片区城市设计，东部新中心以合钢片区为核心，推动老工业基地工业遗产保护与传承，展示工业文明的历史足迹，打造城市青年活力中心；骆岗片区以建设中央公园为核心，打造品质一流的城市新地标、面向全球的国际舞台；空港片区聚力建设先进制造业集聚区，打造联通世界的国际合作示范窗口；三十岗片区聚力建设大科学装置集中区，打造环境清新的最美科研圣地。推进城市“双修”，开展老城更新，重塑城市肌理，让老城区成为延续历史文脉、保留城市记忆的颜值担当。

　　城市建设提品质。开工建设合新高铁、新合肥西站、巢马城际铁路，加快建设合安高铁，积极推进合宁高铁，争取商合杭北段建成通车。加快推进新桥机场二期，力争开工建设通用机场。完成合宁、合芜、合安高速扩容工程，全力实施德上高速合枞段、岳武高速东延无岳段、商宣高速、合六叶高速扩容工程。开工建设合六南通道，加快推进巢庐路、军二路、新蚌埠路等国省干线建设。开通运营轨道交通3号线，加快建设4、5号线和1号线三期工程，积极推进2、3、4号线延长线和6、7、8号线，加快规划S1号线。建成郎溪路高架、裕溪路高架等重点工程，开工建设畅通二环改造工程，加快推进徽州大道南延工程。全力创建公交都市，完善城市慢行系统，推进公共停车场、新能源汽车充电设施建设。提升水电气热等保障能力，完成城市地下综合管廊试点建设任务，加快推进引江济淮工程，开工建设龙河口饮水工程，规划建设巢湖市江水供水工程，实施裕溪一线船闸扩能改造。推深做实“林长制”，精心打造森林长廊示范段，加快实施滁河干渠、环巢湖大道绿化景观提升工程，新增造林5万亩。加快建设城市十大公园、环湖十大湿地和100个小公园、小游园，全面提升城市绿化景观品质，打造更加丰富多彩的美丽空间和市民乐园。

　　城市管理求精细。深化城市管理体制改革，推进重心下移，实现“小网格、大城管、优服务”。巩固城市管理提升年和文明城市创建工作成果，以背街小巷环境卫生整治为重点补齐管理短板。传承城市文化基因，高品质建设一批特色街区，有序整治街道立面景观。完成全国垃圾强制分类试点城市建设任务，提升城乡生活垃圾治理水平。加快打造新型示范性智慧城市，推进数字城管二期建设，建成覆盖全市的数字城管指挥平台。加大信用社会建设力度，全力创成国家信用体系示范城市。

　　（四）高标准实施乡村振兴。坚持农业农村优先发展，编制实施乡村振兴战略规划，推进城乡融合发展，不断提升农业农村现代化水平。

　　着力振兴县域经济。大力建设省级县域特色产业基地，加快县域开发园区优化整合，提升县域经济质量和效益。加大县域经济发展支持力度，加快城市基础设施和优质公共服务供给向农村延伸。肥东、肥西、长丰一体融入主城区，进一步巩固全国百强县位次。庐江、巢湖等高对接主城区，进一步巩固全省十强县位次，全力冲刺全国百强县。

　　着力推进农业现代化。深入推进农业供给侧结构性改革，积极发展“互联网+农业”、智慧农业、休闲农业。大力实施粮食绿色增效工程，推进“粮改经”“粮改饲”“双改单”，新增虾稻共养面积25万亩。深入实施农产品加工六大行动，促进农产品加工向精深转变。完善农业经营体系，构建小农户和现代农业利益联结机制，培育新型农业经营主体1000家以上。制定和实施质量兴农战略规划，推动农村三产深度融合。

　　着力改善农村人居环境。高质量建设一批乡村振兴重点项目，提升农村“双基”水平。推动美丽乡村建设提标扩面，建成89个中心村。深入实施农村人居环境整治三年行动计划，推进农村环境“三大革命”，县域行政村实现环卫治理市场化服务全覆盖，乡镇政府驻地实现污水处理厂设施全覆盖，完成10万户农村改厕任务。深入开展农田水利“最后一公里”建设行动，实施万亩大圩、中小水库和灌区达标工程。全面推进“四好农村路”建设，完成农村道路畅通工程1100公里。大力实施农村土地整治，新增耕地4万亩。强化永久性基本农田用途特殊管制措施，深入开展“大棚房”问题专项整治。积极发展农业循环经济，提升农作物秸秆综合利用水平，深入推进巢湖流域农业面源污染防治。关心农民群众生产生活，确保农村社会和谐稳定。加强乡村社会治理，建设乡风文明、治理有效的现代化农村。

　　（五）高站位推进改革开放。全面深化改革、扩大开放，进一步激发市场活力和内生动力，努力成为引领全省高质量发展的重要动力源。

　　推进重点领域改革。大力推进简政放权，统筹推进“多证合一”和“证照分离”，全力开展“一网一门一次”改革，有效解决市场主体的“难点”“痛点”“堵点”问题，让企业和群众到政府办事像“网购”一样方便。加强政府权力事中事后监管，积极推进跨部门“双随机、一公开”联合抽查。深化国资国企改革，加快推进以管资本为主的国资监管职能转变。深化财税、金融体制改革，全面实施预算绩效管理，规范政府举债融资机制，完善地方金融监管体系。推进农村土地、宅基地“三权”分置和集体产权制度改革，完善盘活农民闲置宅基地和农房政策，稳步开展“三变”改革试点。深化公共资源交易管理体制改革，提升公共资源配置的质量和效率。推进统计体制改革，扎实做好第四次全国经济普查工作。

　　推进区域一体化发展。深度融入长三角一体化发展，全面落实G60科创走廊《松江宣言》，在空间规划、科技创新、产业布局、市场要素、生态环保、基础设施和公共服务等六个方面谋划推进一批重点项目。推进与长江中游城市群互联互通，举办第七届四省会城市会商会。深入落实合肥都市圈一体化发展行动纲要，在城际轨道、产业走廊、环保治理等方面形成一批标志性合作成果，加快打造世界级城市群的区域中心。提升与皖北结对合作水平。

　　扩大对外合作交流。加密中欧班列，加快打造国家级中欧班列集结中心。推进水运港多式联运，促进吞吐量稳定增长。积极申建肉类进境指定口岸，提升航线综合运营水平，谋划开通新的航空货运航线。加快中国（合肥）跨境电商综试区建设，扩大进出口规模。复制推广上海等自贸试验区改革试点经验，积极推动出口加工区升级综合保税区。深入推进中德（合肥）智慧产业园、智能制造国际创新园、“侨梦苑”等建设，打造一批高端国际合作平台。加强与“一带一路”沿线国家和地区务实合作，推进国际友城交流。扎实推进大招商，扩大市产业投资引导基金规模，支持县（市）区、开发区设立专项产业招商基金。实施精准招商、委托招商、产业链招商，全年招商引资增长10%左右。

　　（六）高强度推进“三大攻坚”。坚持抓重点、补短板、强弱项，聚焦解决突出问题，坚决打好三大攻坚关键战役，跨越高质量发展的重大关口。

　　打好防范化解重大风险攻坚战。完善地方金融风险防控机制，建立互联网金融监管长效机制，探索建立重大风险监测预警平台。坚决打击非法集资，开展交易场所清理整顿回头看，强化源头管控，实现共防共打。持续深化政府隐性债务“四清四实”专项整治，降低政府债务风险，增强防范化解风险能力。

　　打好精准脱贫攻坚战。全面落实精准扶贫、精准脱贫基本方略，深入实施脱贫攻坚三年行动，扎实抓好“八大提升”工程，完成建档立卡贫困人口脱贫任务。健全脱贫成果巩固跟踪制度，加强对边缘农户、易返贫农户的跟踪和持续帮扶，有效防止贫困人口返贫。

　　打好污染防治攻坚战。继续聚焦“三大保卫战”和“七大标志性战役”，以最大的决心，下最大的力气，坚定抓好中央和省环保督察问题整改。根据巢湖综合治理绿色发展总体规划，集中打好巢湖水环境治理突击战，统筹推进雨污分流、截污纳管、建厂处理、生态补水、湿地净化等工程，加快实现入湖河流清澈干净，为群众提供更多亲水环境。健全“河长制”“湖长制”，突出治理南淝河，持续巩固双桥河、派河水质达标，确保十五里河消除劣V类，基本消灭城市黑臭水体。启动实施城镇污水处理提质增效三年行动，建成十五里河污水处理厂四期、小仓房污水处理厂三期、陶冲污水处理厂二期等项目，做好雨污管网建设、养护，新建一批城市初期雨水调蓄设施。实施重大专项行动，强化区域联防联控，进一步改善空气质量。严格建设用地和污染地块环境监管，继续推进试点地块土壤修复。严厉打击固体废物非法转移、倾倒、处置行为，加强危险废物处置能力建设。加快构建生态环境监测网络，完善预报预警和质控体系。强化企业环保信用约束。开展各类绿色创建行动，一定要让我们生活的家园天蓝气净、水清岸绿。

　　（七）高水平保障改善民生。把群众关心的关键小事当作政府的头等大事，进一步扩大优质公共服务供给，努力增强人民群众幸福感。

　　提高就业质量和社会保障水平。统筹抓好高校毕业生、农民工、退役军人、城镇困难人员等群体高质量、多渠道、可持续就业，大力援企稳岗，扩大就业容量。加强人力资源市场建设，实施高技能人才培养工程，提升公共就业服务水平。加快建立房地产调控长效机制，构建多层次住房保障体系，发展多元化住房租赁主体，启动9个城中村和危旧小区搬迁改造，综合整治老旧小区122个，新建棚户区改造安置房1.6万套。稳妥推进既有住宅加装电梯工作。完善城镇职工、城乡居民医保政策，进一步提高最低生活保障等社会救助标准。健全养老服务体系，大力推进居家养老、社区养老，加快农村敬老院升级改造，推进市一院、二院、新站高新区老年护理院等项目建设，构建老有所养、老有所依的“快乐老家”。

　　扩大优质教育供给。办好老百姓家门口的学校，推进学前教育普及普惠安全优质发展，统筹城乡义务教育一体化发展，建成中小学26所、幼儿园45所，加快推进合肥六中、合肥九中、市特教中心、合肥幼专新校区等项目建设。扩大中小学午餐服务工程保障范围，多形式试点“课后三点半”服务。大力发展智慧教育，推进家校共育，深入开展新优质学校创建。健全校外培训机构管理长效机制。加大农村教育投入，保障农村教师待遇。加快职教集团公共服务平台建设，深入推进产教融合、校企合作，提升合肥工业学校、经贸旅游学校、黄麓师范学校等办学水平，支持合职院、合肥幼专高水平建设发展。加快推进中德教育合作示范基地建设，支持合肥学院创建合肥大学，支持中科大、合工大、安大建设“双一流”大学。持续推进师德师风建设，全面提升教师队伍整体素质，让最优秀的人为国家培养更优秀的人。

　　推动文化繁荣兴盛。深入践行社会主义核心价值观，进一步提高市民道德水平和人文素养。大力实施全民科学素质行动计划，加快推进市中心图书馆、市科技馆新馆建设，启动建设市美术馆、市青少年活动中心、市妇儿活动中心，完成市博物馆选址立项和方案设计。持续开展全民阅读，新建城市阅读空间30个。引进高层次文艺人才和团队，加大精品创作。实施庐剧、巢湖民歌振兴工程，建立庐剧传承研究中心。坚持政府主导、社会参与，办好合肥国际文博会、上海国际艺术节合肥分会场、中国国际青年电影展等一批国际化、全国性品牌文化活动。扩大群众参与度，开展百场文艺演出进社区、送戏进万村等大众文化活动。

　　推进健康合肥建设。深化医药卫生体制改革，加快紧密型医联（共）体建设，推进公立医院改革、药品供应保障制度改革。改革医保支付方式，探索开展按疾病诊断分组、慢病管理付费。积极发展“互联网+医疗健康”，推进国家健康医疗大数据中部中心及产业园、全民健康信息平台、智慧医院建设。加快推动市三院新区、市空港医院、市中医院、市中心血站等项目建设。做实家庭医生签约服务，推进落实分级诊疗。积极创建国家全民运动健身模范市，继续建设一批群众身边的多样化体育设施。

提升合肥国际马拉松等重大赛事水平，举办首届全民健身运动会。全力创建国家食品安全示范城市，完成城区存量菜市场改造提升，切实保障人民群众“舌尖上的安全”。

　　加强和创新社会治理。深入开展“七五”普法，努力实现全国普法先进城市“四连冠”。加强信访法治化建设，提升访调对接水平，预防和化解社会矛盾。加快街道、社区“两级中心”建设，打造一批智慧社区，构建居民“十五分钟”生活圈。加强互联网建设与管理，营造和谐文明网络空间。持续开展扫黑除恶，完善立体化社会治安防控体系，全面净化社会治安环境。推进应急管理体制改革，提高城市安全运行精细化管理能力，促进安全生产形势持续稳定。推进军民融合深度发展，努力争创全国双拥模范城“九连冠”。支持工会、共青团、妇联、红十字会等人民团体广泛参与社会治理和公共服务，扎实做好民族宗教、对台侨务、人民防空、防震减灾、残疾人、地方志、档案、保密、社科、仲裁等工作。

　　三、加强政府自身建设

　　开启新征程，完成新目标，对政府自身建设提出了新的更高要求。我们要树立新气象，展现新作为，不断提升政府治理能力现代化水平。

　　锻造忠诚可靠的政治品格。始终树牢“四个意识”，切实增强“四个自信”，坚决做到“两个维护”，在思想上政治上行动上同以习近平同志为核心的党中央保持高度一致。认真开展“不忘初心、牢记使命”主题教育，巩固提升“讲严立”专题警示教育成果，持续开展“三查三问”，坚决把党中央国务院、省委省政府和市委决策部署不折不扣落到实处。坚持用习近平新时代中国特色社会主义思想武装头脑，全面增强“八种本领”，在学习和实践中理思路、找方法，强化专业思维、提高专业素质，善于处理各种复杂矛盾，牢牢把握工作主动权。

　　坚守执政为民的初心使命。始终把人民放在心中最高位置，带头深入一线、深入基层，及时了解群众所思所盼所想，一件一件办好老百姓的身边小事、急事、难事。加快转变政府职能，完成新一轮机构改革各项任务。深入推进“互联网+政务服务”，大力推行“不见面审批”。聚焦群众最关心最直接最现实的利益问题，扎实开展为民办实事，在改革发展中补齐民生短板，在高质量发展中增进民生福祉， 让人民群众生活得更安心、更省心、更舒心。

　　筑牢依法行政的工作理念。始终坚持厉行法治、规范用权，深入推进法治政府建设，严格遵守宪法和法律，自觉运用法治思维、法治方式开展工作，努力把政府各项工作纳入法治化轨道。认真执行市人大及其常委会的决议决定，自觉接受市人大的法律监督和工作监督，主动接受市政协的民主监督，广泛听取各民主党派、工商联、无党派人士和人民团体意见，高质量办理人大代表议案建议和政协提案。全面落实合法性审查、风险评估等决策程序，不断提升行政决策科学化、民主化、法治化水平。

　　营造风清气正的政治生态。始终坚持全面从严治党，认真履行主体责任，严格遵守《纪律处分条例》。持续推进审计全覆盖，加快构建集中统一、全面覆盖、权威高效的审计监督体系。深入推进政府系统党风廉政建设，严厉查处损害群众利益的不正之风和腐败行为。深入贯彻中央八项规定精神及省、市委实施细则，严厉整治形式主义、官僚主义，坚持不懈纠“四风”、正作风。带头执行《廉洁自律准则》，坚持用制度管权管事管人，严格控制“三公”经费，真正做到干部清正、政府清廉、政治清明。

　　弘扬担当作为的精神风尚。始终坚持崇尚实干、勇于担当，不驰于空想，不骛于虚声，说实话、谋实事、出实招、求实效。严格执行工作责任制，构建科学有效的考核机制，强化督查问责，深化效能建设，严厉整肃庸政懒政怠政行为。健全激励机制和容错纠错机制，给干事者鼓劲，为担当者撑腰，让广大公务人员以实干推动发展、赢得未来，努力递交让人民满意的时代答卷。

　　各位代表！

　　奔跑在奋斗征程，追梦于美好时代。让我们紧密团结在以习近平同志为核心的党中央周围，在省委省政府和市委的坚强领导下，开拓创新，奋发有为，加快打造具有国际影响力的创新之都，不断谱写社会主义现代化建设合肥篇章，为建设现代化五大发展美好安徽作出新的更大贡献，以优异成绩向新中国成立70周年献礼！
